# Kaposi's sarcoma associated herpes virus-encoded viral FLICE inhibitory protein activates transcription from HIV-1 Long Terminal Repeat via the classical NF-κB pathway and functionally cooperates with Tat

**DOI:** 10.1186/1742-4690-2-9

**Published:** 2005-02-15

**Authors:** Qinmiao Sun, Hittu Matta, Preet M Chaudhary

**Affiliations:** 1 Hamon Center for Therapeutic Oncology Research, UT Southwestern Medical Center, Dallas TX 75390-8593, USA; 2 Department of Medicine, Division of Hematology-Oncology and the Hillman Cancer Center, University of Pittsburgh, PA 15213, USA

## Abstract

**Background:**

The nuclear transcription factor NF-κB binds to the HIV-1 long terminal repeat (LTR) and is a key regulator of HIV-1 gene expression in cells latently infected with this virus. In this report, we have analyzed the ability of Kaposi's sarcoma associate herpes virus (KSHV, also known as Human Herpes virus 8)-encoded viral FLIP (Fas-associated death domain-like IL-1 beta-converting enzyme inhibitory protein) K13 to activate the HIV-1 LTR.

**Results:**

We present evidence that vFLIP K13 activates HIV-1 LTR via the activation of the classical NF-κB pathway involving c-Rel, p65 and p50 subunits. K13-induced HIV-1 LTR transcriptional activation requires the cooperative interaction of all three components of the IKK complex and can be effectively blocked by inhibitors of the classical NF-κB pathway. K13 mutants that lacked the ability to activate the NF-κB pathway also failed to activate the HIV-1 LTR. K13 could effectively activate a HIV-1 LTR reporter construct lacking the Tat binding site but failed to activate a construct lacking the NF-κB binding sites. However, coexpression of HIV-1 Tat with K13 led to synergistic activation of HIV-1 LTR. Finally, K13 differentially activated HIV-1 LTRs derived from different strains of HIV-1, which correlated with their responsiveness to NF-κB pathway.

**Conclusions:**

Our results suggest that concomitant infection with KSHV/HHV8 may stimulate HIV-1 LTR via vFLIP K13-induced classical NF-κB pathway which cooperates with HIV-1 Tat protein.

## Background

The human immunodeficiency virus type 1 (HIV-1) establishes latent infection following integration into the host genome [1]. The expression of integrated HIV-1 provirus in cells latently infected with this virus is controlled at the level of transcription by an interplay between distinct cellular and viral transcription factors which bind to the HIV-1 long terminal repeat (LTR) [1-4]. The HIV-1 LTR is divided into three regions: U3, R and U5, which contain four functional elements: transactivation response element (TAR), a basal or core promoter, a core enhancer, and a modulatory element [1,4]. The viral transactivator Tat is a key activator of HIV-1 LTR via its binding to the TAR region, while the core region contains three binding sites for Sp1 transcription factor and a TATA box [1]. The enhancer region of HIV-1 LTR contains two highly conserved consecutive copies of κB elements at nucleotides -104 to -81 that are critical for HIV-1 replication in T cells [1]. Finally, the modulatory region harbors binding sites for numerous transcription factors, such as c-Myb, NF-AT, USF and AP1. Among the various signaling pathways known to activate HIV-1 LTR, the NF-κB pathway is particularly important as it is activated by several cytokines involved in immune and inflammatory response [1]. However, all pathways that stimulate NF-κB do not reactivate latent HIV and HIV-1 gene expression is also known to be regulated by NF-κB-independent mechanisms, for example via Tat [2,3].

There are five known members of the NF-κB family in mammalian cells including p50/p105 (NF-κB1), p52/p100 (NF-κB2), p65 (RelA), c-Rel, and RelB [5,6]. Although many dimeric forms of NF-κB have been described, the classical NF-κB complex is a heterodimer of the p65/RelA and p50 subunits. The activity of NF-κB is tightly regulated by their association with a family of inhibitory proteins, called IκBs [5-7]. The best characterized Rel-IκB interaction is between IκBα and p65-p50 dimer, which blocks the ability of NF-κB to enter the nucleus. Stimulation by a number of stimuli results in the activation of a multi-subunit IκB kinase (IKK) complex, which contains two catalytic subunits, IKK1/IKKα and IKK2/IKKβ, and a regulatory subunit, NEMO/IKKγ [7]. The IKK complex leads to the inducible phosphorylation of IκB proteins at two conserved serine residues located within their N-terminal region [5]. Phosphorylation of IκB proteins lead to their ubiquitination and subsequent proteasome-mediated degradation, thereby releasing NF-κB from their inhibitory influence [7]. Once released, NF-κB is free to migrate to the nucleus and bind to the promoter of specific genes possessing its cognate binding site. In addition to the above classical NF-κB pathway, an alternative (or noncanonical) pathway of NF-κB activation that involves proteasome-mediated processing of p100/NF-κB2 into p52 subunit, has been described recently [8]. Unlike the classical NF-κB pathway, which involves IKK2 and NEMO, activation of the alternative NF-κB pathway by TNF family receptors is critically dependent on NIK and IKK1 [9,10].

Kaposi's sarcoma associated herpes virus (KSHV), also known as Human herpes virus 8 (HHV8), is a γ-2 herpes virus which is frequently associated with malignancy among AIDS patients [11-13]. In addition to Kaposi's sarcoma (KS), KSHV genome has been consistently found in primary effusion lymphoma (PEL) or body cavity lymphoma and multicentric Castleman's disease. KSHV genome is known to encode for homologs of several cytokines, chemokines and their receptors [11-13]. However, none of the above proteins is expressed in cells latently-infected with KSHV [11]. KSHV also encodes for a protein called K13 (or orf71), which is one of the few viral proteins known to be expressed in cells latently infected with KSHV [11,14-16].

The K13 protein contains two homologous copies of a Death Effector Domain (DED) that is also present in the prodomains of caspase 8 (also known as FLICE), caspase 10 and cellular FLICE Inhibitory Protein (cFLIP, also known as MRIT) [17]. Proteins with two DEDs have been discovered in other viruses as well, including MC159L and MC160 from the molluscum contagiosum virus and E8 from the equine herpes virus 2 [18-20]. These virally encoded DED-containing proteins are collectively referred to as vFLIPs (viral FLICE Inhibitory Proteins) [18-20].

We recently demonstrated that KSHV vFLIP K13 possesses the unique ability to activate both the classical and the alternate NF-κB pathways [21-24]. Several recent studies suggest that binding of NF-κB to HIV-1 LTR may not be sufficient and interaction with additional viral and cellular factors may be required to induce its transcriptional activation [25,26]. As such, in this report we have carried out a detailed analysis of the ability of K13 to activate the HIV-1 LTR and analyzed the contribution of the canonical *vs *alternate NF-κB signaling pathways, various subunits of the IKK complex and the HIV-1 Tat to this process.

## Results

### vFLIP K13 activates the HIV-1 LTR

**Figure 1:**
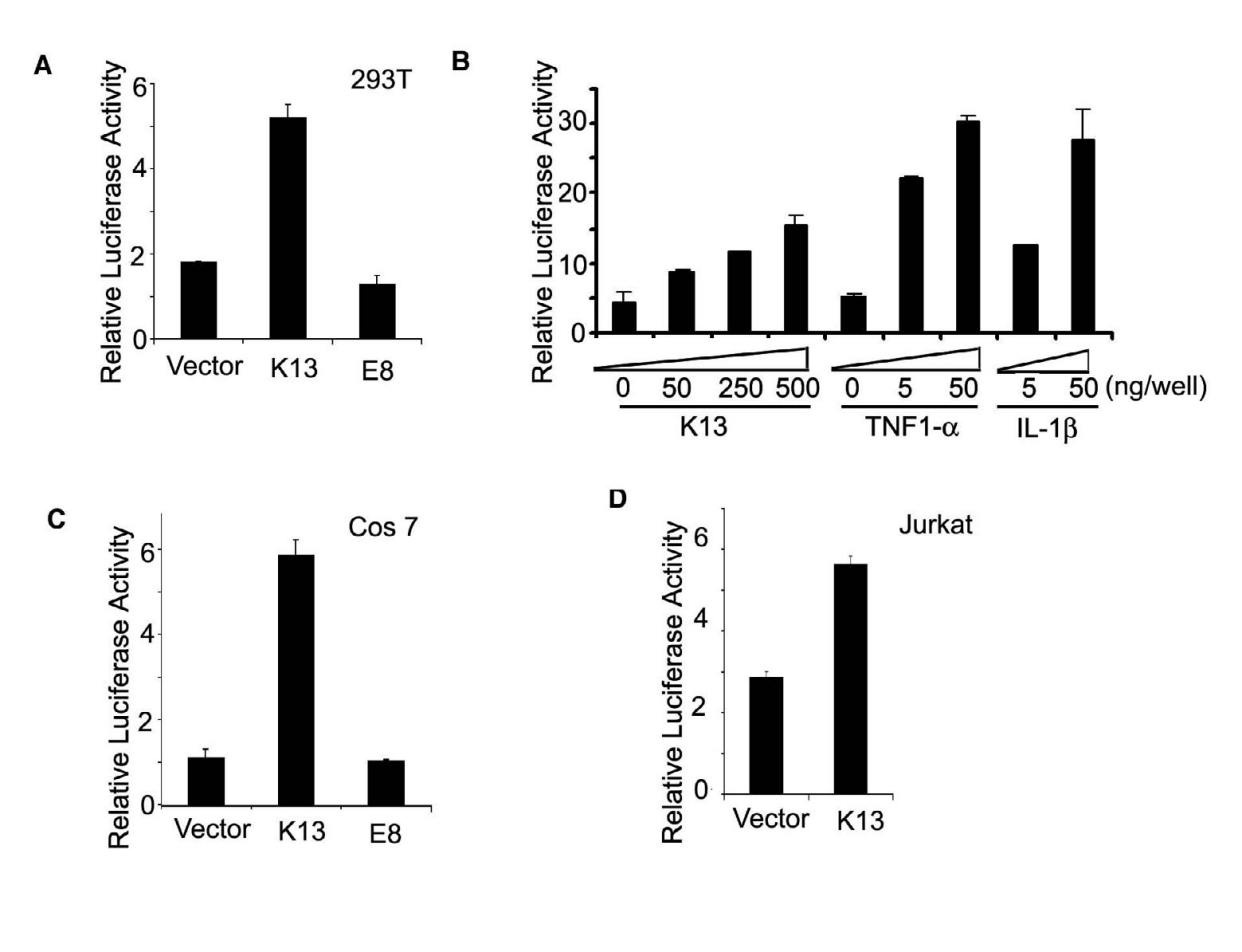
**K13 activates HIV-1 LTR promoter**. A. 293T cells were transfected with an empty vector or the indicated constructs (100 ng/well) along with an HIV-1 LTR/luciferase reporter construct (10 ng/well) and a pRSV/LacZ (β-galactosidase) reporter construct (75 ng/well), and the experiment was performed as described under "Materials and Methods." The values shown are averages (Mean ± S.E.) of one representative experiment out of three in which each transfection was performed in duplicate. B. A dose-response analysis of HIV-1 LTR activation by K13 and pro-inflammatory cytokines. 293T cells were transfected with the indicated amounts of a K13 expression plasmid and luciferase assay performed 36 h post-transfection as described for (A). The total amount of transfected DNA was kept constant by adding an empty vector. For experiments involving TNF-α and IL-1β, cells were treated with the indicated concentration of cytokines 12 h after transfection of the reporter plasmids and assayed for reporter activity after 24 h of stimulation. C. K13 activates HIV-1 LTR in Cos-7 cells. The experiment was performed as described in 1A except LIPOFECTAMINE 2000 Reagent (Invitrogen, Carlsbad, CA) was used for transfection and Renilla luciferase was used for normalization. D. K13 activates HIV-1 LTR in Jurkat cells. The experiment was performed as described for 1C by using LIPOFECTAMINE 2000 Reagent (Invitrogen, Carlsbad, CA).

We used a luciferase reporter construct to test the effect of vFLIP K13 on HIV-1 LTR transcriptional activation. This reporter construct expresses the firefly luciferase gene downstream of the HIV-1 LTR. As shown in Fig. 1A–C transient transfection of vFLIP K13 in 293T and Cos7 cells led to significant (3 and 5 fold, respectively) activation of the HIV-1 LTR where as expression of the vFLIP E8 from the equine herpes virus 2 failed to do so. As HIV-1 LTR is known to be responsive to proinflammatory cytokines, we also carried out a comparative analysis of the HIV-1 LTR activation by K13, TNF-α and IL-1β in 293T cells. As shown in Fig 1C, while K13-induced approximately 3-fold increase in HIV-1 LTR transcriptional activation, treatment with TNF-α(50 ng/ml) and IL-1β (50 ng/ml) resulted in 5–6 fold increase. A possible explanation for this difference lies in the fact that unlike TNF-α and IL-1β, K13 lacks the ability to induce the transcription factor AP1, which is known to activate HIV-1 LTR. We also tested whether vFLIP K13 possesses the ability to activate the HIV-1 LTR in cells naturally infected with HIV-1. As shown in Fig. 1D, transient transfection of K13 in Jurkat cells (human T cell lymphoma cell line) led to modest (2-fold) activation of HIV-1 LTR transcription activity.

### K13 mutants defective in NF-κB activation fail to activate HIV-1 LTR

**Figure 2:**
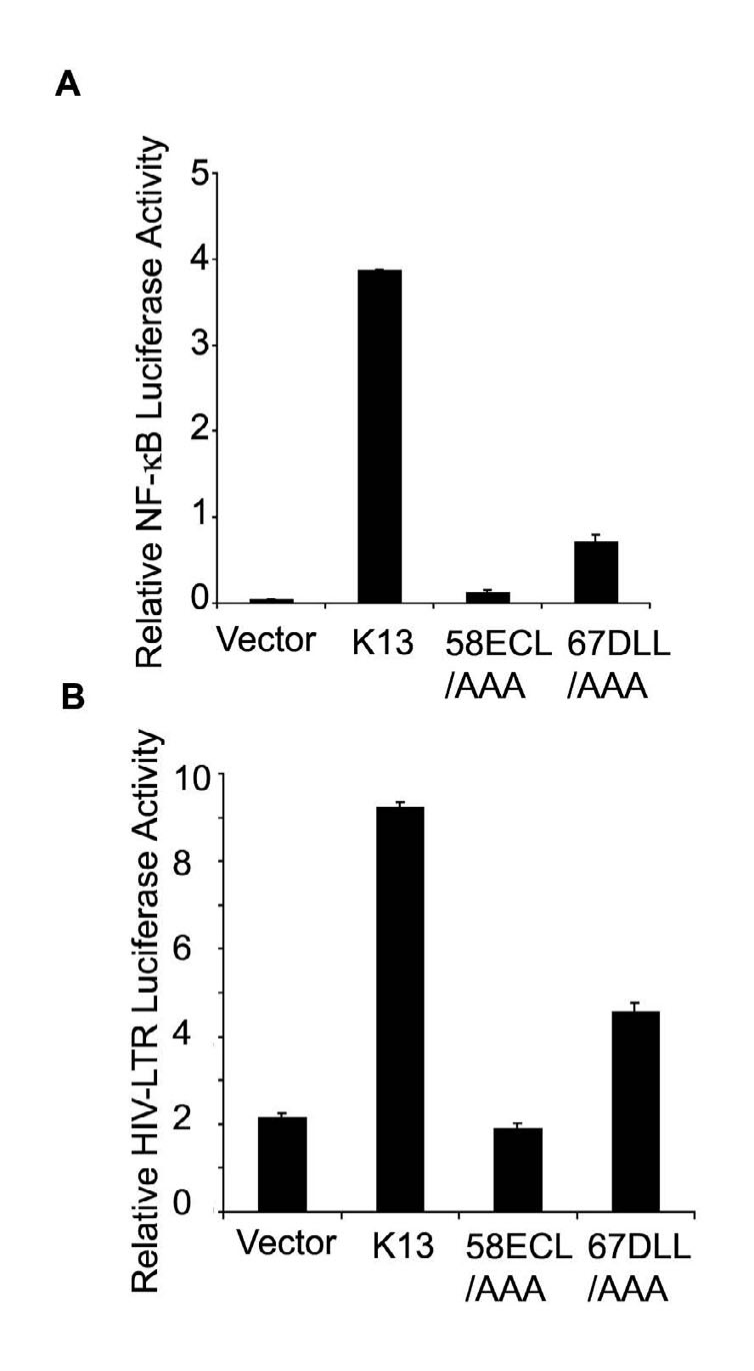
**Activation of HIV- LTR by K13 mutants correlates with their ability to activate the NF-κB pathway**. A. NF-κB activation by mutants of K13. 293T cells were transfected with an empty vector (pCDNA3) or the indicated K13 expression constructs (100 ng/well) along with an NF-κB/luciferase reporter construct (75 ng/well) and an pRSV/LacZ (β-galactosidase) reporter construct (75 ng/well) and luciferase reporter assay performed as described in Fig. 1A. The values shown are averages (mean ± SEM) of one representative experiment out of three in which each transfection was performed in duplicate. B. HIV-1 LTR activation by wild-type and mutant K13 constructs. The experiment was performed as described for Fig. 1A.

We have recently generated point mutants of the vFLIP K13 which differ in their ability to activate the NF-κB pathway [27]. In order to test the hypothesis that vFLIP K13 activates the HIV-1 LTR via NF-κB pathway, we carried out a comparative analysis of the ability of wild-type and mutant K13 constructs to activate the HIV-1 LTR reporter construct. In a parallel experiment, we also tested the effect of different K13 constructs on an NF-κB luciferase reporter construct to serve as a positive control. The luciferase expression in the latter construct is driven by four copies of a consensus NF-κB binding-site [28]. Consistent with our published results [27], the triple mutant 58AAA demonstrated a complete lack of NF-κB reporter activation while the mutant 67AAA retained partial ability to do so (Fig 2A). Importantly, essentially a similar pattern of reporter activation was obtained when the wild-type and mutant K13 constructs were tested on the HIV-1 LTR reporter construct (Fig 2B). Collectively, the above results suggested the involvement of the NF-κB pathway in vFLIP K13-induced HIV-1 LTR activation.

### vFLIP K13 induces binding of specific transcription factors to HIV-1 LTR

**Figure 3:**
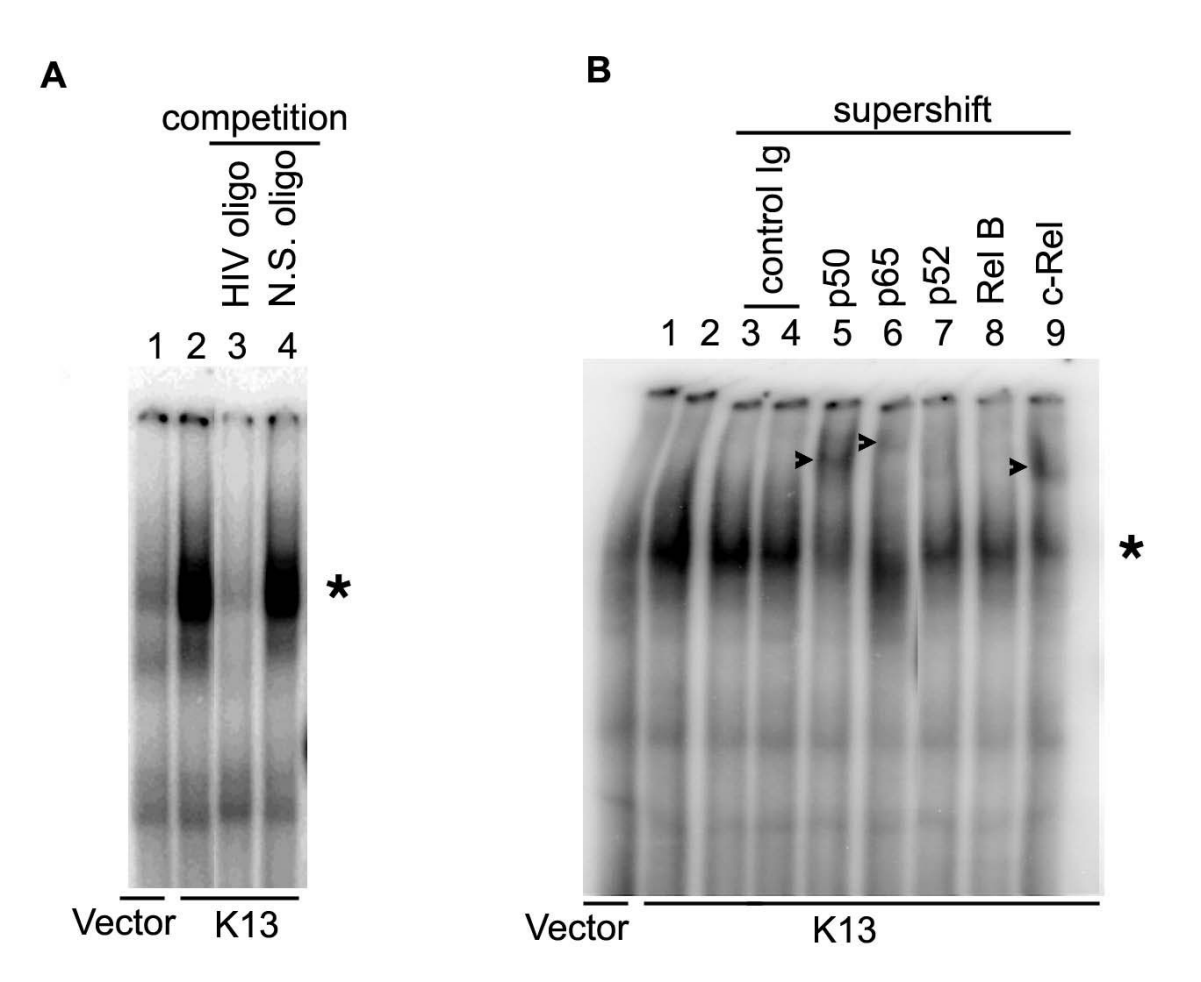
**Electrophoretic mobility shift assay**. A. The nuclear extract from Jurkat cells stably expressing an empty vector (lane 1) and K13 (lanes 2–4) were used for EMSA. The position of the induced HIV-1 LTR complex is marked with an asterisk. The specificity of the complex is demonstrated by competition with excess cold HIV-1 LTR probe (lane 3) and a nonspecific (N.S.) probe (lane 4), respectively. B. A supershift assay showing the subunit composition of K13-induced NF-κB subunits bound to HIV-1 LTR. The supershift assay was performed using a control rabbit antisera (lane 3), control mouse antisera (lane 4), or antisera against p50 (lane 5), p65 (lane 6), p52 (lane 7), Rel B (lane 8) and c-Rel (lane 9) subunits of NF-κB, respectively. The position of the induced HIV-1 LTR complex is marked with an asterisk, while the super-shifted bands are marked by arrowheads.

In order to test the hypothesis that vFLIP K13 activates HIV-1 LTR by inducing the binding of specific transcription factors to the NF-κB binding sites present in the HIV-1 LTR, we used an electrophoretic mobility shift assay (EMSA). As shown in Fig. 3A, nuclear extracts from Jurkat cells expressing vFLIP K13 demonstrated significant DNA-binding activity on radiolabelled oligonucleotides-derived from the NF-κB binding sites present in HIV-1 LTR. In contrast, no HIV-1 LTR DNA-binding activity was observed in nuclear extracts of empty vector-expressing cells (Fig. 3A, compare lanes 1 and 2). The specificity of the complex was demonstrated by its disappearance upon competition with excess cold HIV-1 LTR oligonucleotide duplex and lack of effect upon competition with a non-specific oligonucleotide duplex (Fig. 3A, lanes 3 and 4).

### Nature and subunit composition of K13-induced transcription factors bound to HIV-1 LTR

In addition to the classical NF-κB pathway, an alternative (or non-canonical) pathway of NF-κB activation, which involves proteasome-mediated processing of p100/NF-κB2 into p52 subunit, has been described [8]. We have recently demonstrated that vFLIP K13 can activate the alternate NF-κB pathway via an IKK1-dependent and NIK- and IKK2-independent process [24]. In order to determine the contribution of the classical *vs *alternate NF-κB pathway to vFLIP K13-induced HIV-1 LTR activation, we used a supershift assay to analyze the nature of the protein complexes bound to HIV-1 LTR from nuclear extracts of vFLIP K13-expressing cells. This assay demonstrated that p50 and c-Rel subunits are the major components of the HIV-1 LTR-bound NF-κB complexes induced by vFLIP K13 with modest contribution from the p65 subunit (Fig. 3B). As the p50, c-Rel and p65 subunits are primarily activated by the classical NF-κB pathway, the above results support the hypothesis that K13 activates the HIV-1 LTR via the classical NF-κB pathway.

### Role of classical NF-κB activation in K13-induced HIV-1 LTR reporter activity

**Figure 4:**
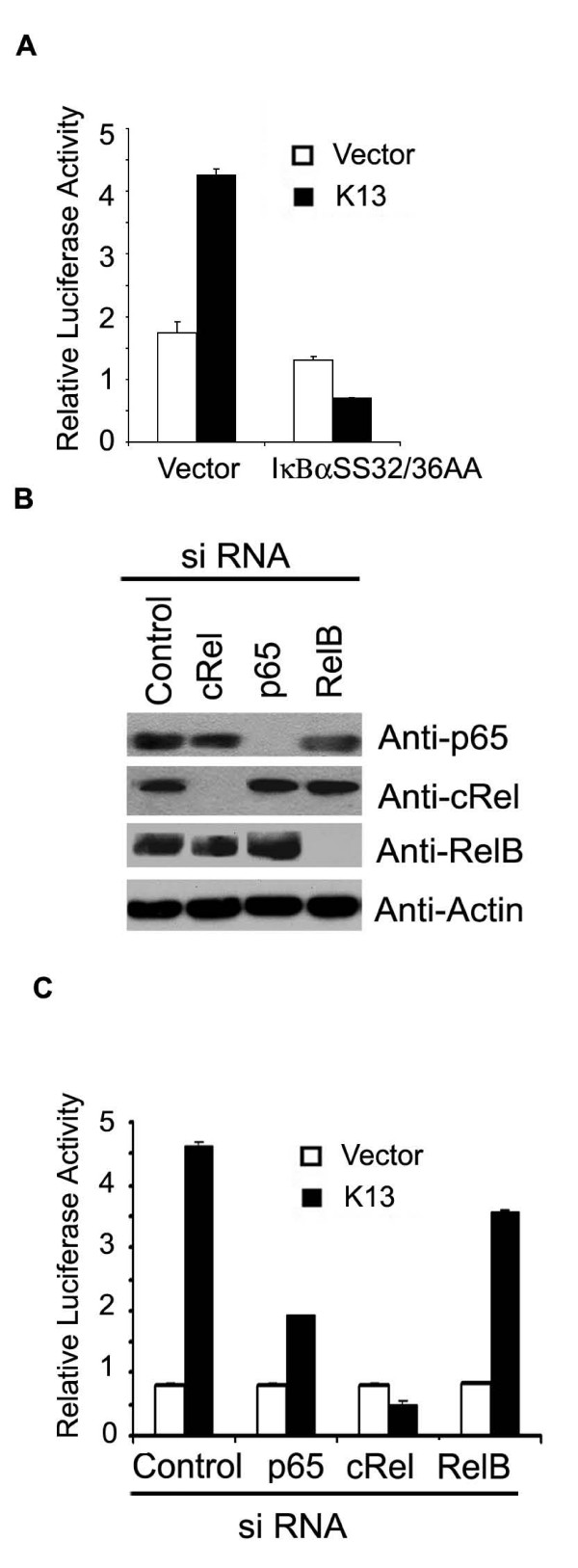
**K13 activates HIV LTR through the classical NF-κB pathway**. A. 293T cells were transfected with an empty vector or K13 along with an HIV LTR/luciferase reporter construct and a β-galactosidase reporter construct as described in Fig. 1A. The amount of IκBαSS32/36AA inhibitor plasmid (500 ng/well) was five times the amount of vector (pcDNA3) or K13 (100 ng/well) plasmid. The values shown are averages (Mean ± S.E.) of one representative experiment out of three in which each transfection was performed in duplicate. B. Western blot analysis showing siRNA-mediated knock-down of p65, c-Rel and RelB expression. The blot was re-probed with a monoclonal antibody against actin (bottom panel) to show equal loading of all lanes and specificity of gene silencing. C. 293T cells were transfected with an empty vector or a K13 expression plasmid along with a control siRNA oligo-duplexes or siRNA duplexes against c-Rel, p65 and RelB, respectively. The luciferase reporter assay was performed as described in Fig 1A.

We have previously demonstrated that vFLIP K13 activates the classical NF-κB pathway via phosphorylation of IκBα, which leads to its ubiquitination and subsequent degradation via proteasome [22]. We used a phosphorylation-resistant mutant of IκBα to test the involvement of the classical NF-κB pathway in vFLIP K13-induced HIV-1 LTR reporter activity. As shown in Fig. 4A, a phosphorylation-resistant mutant of IκBα (IκBαSS32/36AA), in which the two critical N-terminal serine residues have been mutated to alanine, completely blocked vFLIP K13-induced HIV-1 LTR reporter activity.

We used siRNA-mediated downregulation of key subunits of the classical and alternate NF-κB pathways to test their involvement in K13-induced HIV-1 LTR activation. As shown in Fig. 4B, we achieved effective silencing of c-Rel and RelA/p65 expression by siRNA-mediated silencing. Consistent with our supershift assay (Fig. 3B), siRNA-mediated silencing of c-Rel expression led to almost complete suppression of K13-induced HIV-1 LTR activation (Fig. 4C). Similarly, silencing of p65 expression led to significant suppression of HIV-1 LTR activity, although some residual activity was still evident (Fig. 4C). Although p100 acts as a precursor of p52, another important function of p100 is to retain the RelB/p50 and RelB/p52 complexes in the cytoplasm. As such, in order to shut-off the alternate NF-κB pathway, we chose to silence the expression of RelB. As shown in Fig. 4B–C, siRNA-mediated downregulation of RelB, had no significant effect on K13-induced HIV-1 LTR activity. We also failed to observe any effect of p100/p52 silencing on HIV-1 LTR activation (data not shown). Taken together, the above results demonstrate a key role of the c-Rel and p65 subunits of the classical NF-κB pathway in K13-induced HIV-1 LTR reporter activation.

### Role of individual subunits of the IKK complex in K13-induced HIV-1 LTR activation

**Figure 5:**
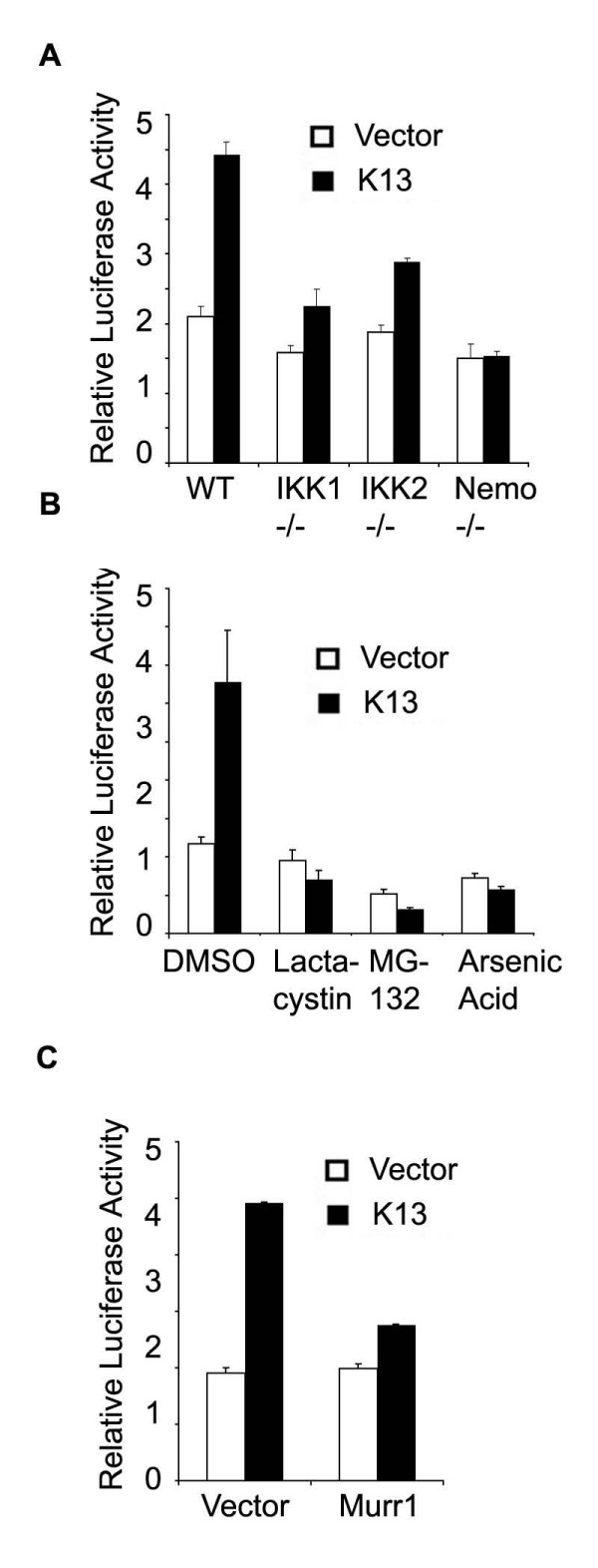
**Mechanism of K13-induced HIV-1 LTR activation**. A. Role of IKK complex in K13-induced HIV-1 LTR reporter activity. Wild-type and IKK-deficient cells were transiently transfected with an empty vector or K13 expression plasmid (500 ng/well) along with an HIV/luciferase reporter construct (100 ng/well) and a synthetic Renilla luciferase (phRL-TK) reporter vector (75 ng/well) by using LIPOFECTAMINE 2000 Reagent (Invitrogen, Carlsbad, CA) according to manufacturer's instruction. Thirty-six hours after transfection, cell lysates were used for reporter assays. Luciferase activity was normalized relative to the Renilla luciferase activity to control for the difference in the transfection efficiency. The values shown are averages (Mean ± S.E.) of one representative experiment out of three in which each transfection was performed in duplicate. B. Inhibitors of the NF-κB pathway significantly block K13-actived HIV LTR promoter. 293T cells were transfected with the empty vector or K13 along with an HIV LTR/luciferase reporter construct and a β-galactosidase reporter construct as described in Fig.1A. Eight hours after transfection, cells were treated with DMSO or different inhibitors for 24 h and then lysed for the reporter assay. The values shown are averages (Mean ± S.E.) of one representative experiment out of three in which each transfection was performed in duplicate. C. Effect of Murr1 on K13-induced HIV-1 LTR activation. 293T cells were transfected with an empty vector (pCDNA3) or K13 along with an HIV LTR/luciferase reporter construct and a β-galactosidase reporter construct as described in Fig. 1A. The amount of Murr1 plasmid (500ng/well) was five times the amount of vector or K13 (100ng/well). The values shown are averages (Mean ± S.E.) of one representative experiment out of three in which each transfection was performed in duplicate.

K13 is known to associate with a 700 kDa multi-subunit IKK complex, which consists of two catalytic subunits, IKK1/IKKα and IKK2/IKKβ and a regulatory subunit, NEMO/IKKγ [22]. We tested the involvement of the individual components of the IKK complex in vFLIP K13-induced HIV-1 LTR reporter activity by using mouse fibroblast (MEF) cells deficient in IKK1, IKK2 and NEMO, respectively. As shown in Fig. 5A, we observed significant HIV-1 LTR reporter activity by the expression of vFLIP K13 in the wild type MEF cells. In contrast, almost no HIV-1 LTR reporter activity was observed in NEMO-deficient cells. However, some residual HIV-1 LTR reporter activity was observed in IKK1- and IKK2-deficient MEF cells. Collectively, the above results suggest that synergistic action of IKK1, IKK2 and NEMO is required for maximal activation of HIV-1 LTR by K13.

Next we sought to determine whether pharmacological inhibitors of the NF-κB pathway may be used to block vFLIP K13-induced HIV-1 LTR reporter activation. Lactacystin and MG132 are inhibitors of proteasome and block the NF-κB pathway by preventing the degradation of IκB. On the other hand, arsenic acid is believed to block the NF-κB pathway by inhibiting the IKK complex [29]. As shown in Fig. 5B, vFLIP K13-induced HIV-1 LTR reporter activation was effectively blocked by MG132, lactacystin and arsenic acid. These results suggest that inhibitors of the NF-κB pathway might have a role in preventing K13-induced HIV-1 LTR reporter activation.

### Effect of Murr1 on K13-induced HIV-1 LTR activation

Murr1 is a gene product that has been previously implicated in copper regulation [30,31]. A recent study demonstrated that Murr1 is highly expressed in CD4+ T cells and serve as a genetic inhibitor factor for HIV-1 replication in the resting lymphocytes [32]. Murr1 was shown to block HIV-1 LTR activation and HIV-1 replication by inhibiting the proteasomal degradation of IκB and blocking basal and cytokine-stimulated NF-κB activation [32]. Based on the above study demonstrating the importance of Murr1 as an endogenous regulator of HIV-1 LTR activation, we tested its effect on K13-induced HIV-1 LTR activation. As shown in Figure 5C, co-expression of Murr1 led to significant block in K13-induced HIV-1 LTR reporter activity, thereby suggesting that K13-induced activation of HIV-1 replication in resting lymphoid cells may be regulated by Murr1 and K13 may selectively activate HIV-1 replication in activated cells in which expression of Murr1 is known to be down-regulated [32].

### Synergistic activation of HIV-1 LTR by vFLIP K13 and HIV Tat protein

**Figure 6:**
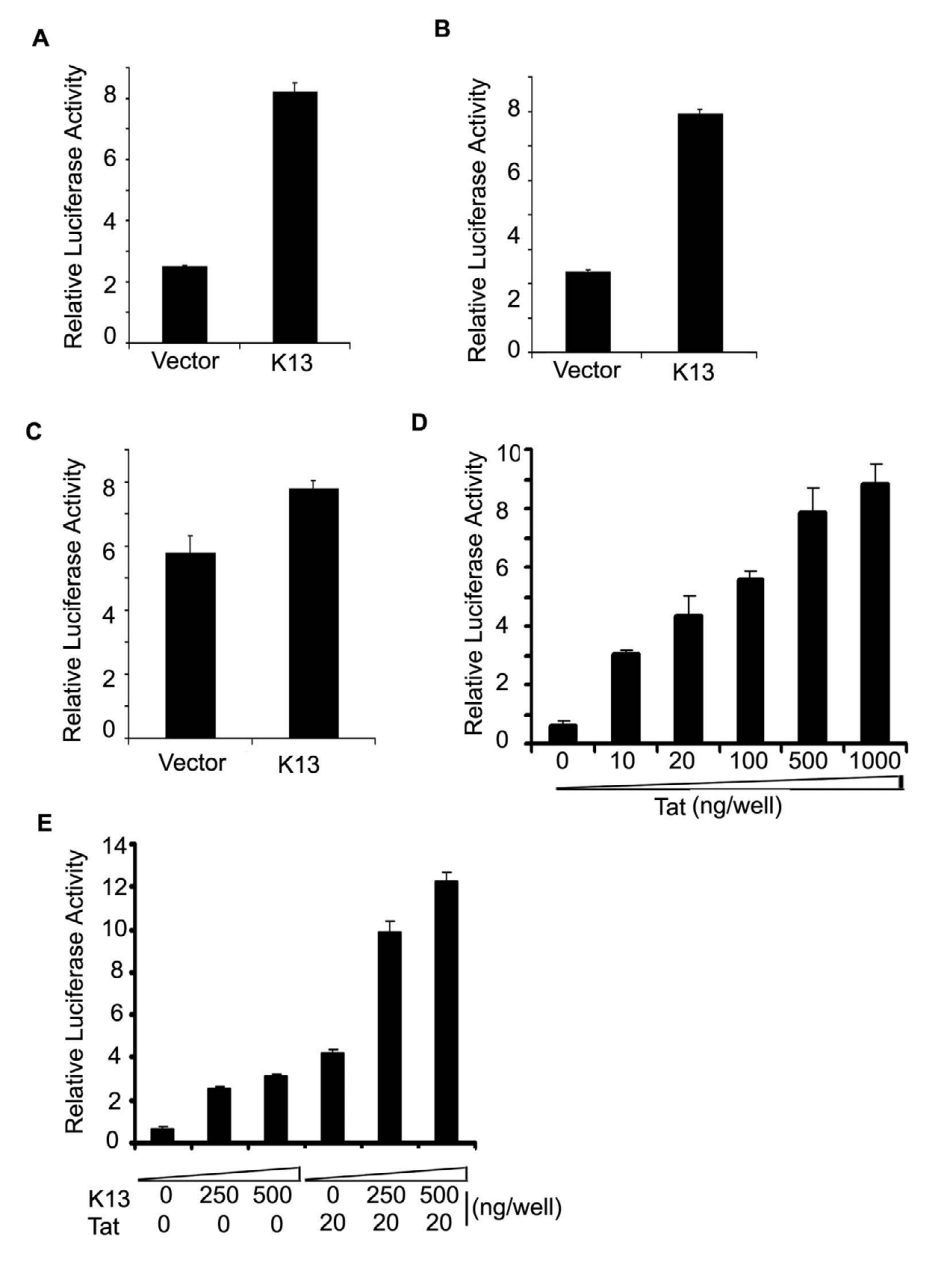
**Effect of HIV Tat protein on K13-induced HIV-1 LTR activation**. A-C. 293T cells were transfected with an empty vector (pCDNA3) or K13 along with different HIV LTR/luciferase reporter constructs and β-galactosidase as described in Fig. 1A. The values shown are averages (Mean ± S.E.) of one representative experiment out of three in which each transfection was performed in duplicate. A, Wild type HIV-1 LTR reporter; B, HIV-1 LTR reporter with deletion of Tat-binding site (nucleotides +23 to +25); C. HIV-1 LTR reporter lacking the NF-κB binding-sites. D. Dose-response analysis of Tat-induced HIV-1 LTR activation. E. K13 and Tat synergistically activate HIV-1 LTR. The experiment was performed as described for Fig. 6 A. The total amount of transfected DNA was kept constant by adding empty vector.

HIV-1 Tat is a viral nuclear protein that plays an essential role in HIV-1 gene expression at the transcriptional level [2,3]. Tat has been shown to associate with p300/CBP and P/CAF histone acetyltransferases (HAT) and efficient activation of the integrated HIV-1 LTR is largely dependent on Tat-dependent rearrangement of the nucleosome positioned at the transcription start site [2]. HIV-1 LTR is known to bind and respond to HIV Tat protein via a specific Tat-binding site [2]. We used deletion mutagenesis of the HIV-1 LTR to test whether vFLIP induced transcriptional activation is dependent on this Tat-binding site. As shown in Figures 6A and 6B, a bulge mutant (containing deletion of nucleotides +23/+25) of HIV-1 LTR, which is defective in Tat activation [33], had no significant effect on vFLIP K13-induced reporter activity. In contrast, vFLIP K13 failed to activate a luciferase report construct containing an HIV-1 LTR in which the NF-κB binding sites had been mutated (Fig. 6C). The above results confirm that vFLIP activates the HIV-1 LTR via the NF-κB binding sites and can do so independent of the Tat-binding site.

Transcriptional activation of genes is usually regulated by multiple transcription factors acting in concert. Thus, while NF-κB has been shown to play a major role in the activation of the HIV-1 LTR, it fails to do so when acting alone [25,34-36]. Along the same lines, the transactivating function of Tat protein requires the presence of NF-κB sites in the HIV-1 LTR and Tat protein is known to cooperate with NF-κB to activate the HIV-1 LTR [1,34,36]. We hypothesized that a functional interaction between K13-induced NF-κB and Tat may be particularly important in the early stages of HIV-1 infection when the amount of Tat is limited. To test this hypothesis, we began by performing a dose-response analysis of Tat and selected a dose of Tat (20 ng/ml) which led to sub-maximal activation of HIV-1 LTR activation in 293T cells (Fig. 6D). Next, we analyzed the effect of co-expression of Tat on K13-induced HIV-1 LTR activation. As shown in Figure 6E, while transfection of K13 (250–500 ng/well) led to approximately 2.5–3.5 fold increase in HIV-1 LTR activation, transfection of Tat (20 ng/well) induced 4-fold increase in HIV-1 LTR activity. However, co-expression of K13 with Tat led to a synergistic 12-fold activation of the HIV-1 LTR. These results suggest that K13-induced NF-κB functions synergistically with the Tat protein to activate the HIV-1 LTR.

### Effect of vFLIP K13 on LTRs-Derived from different strains of HIV

**Figure 7:**
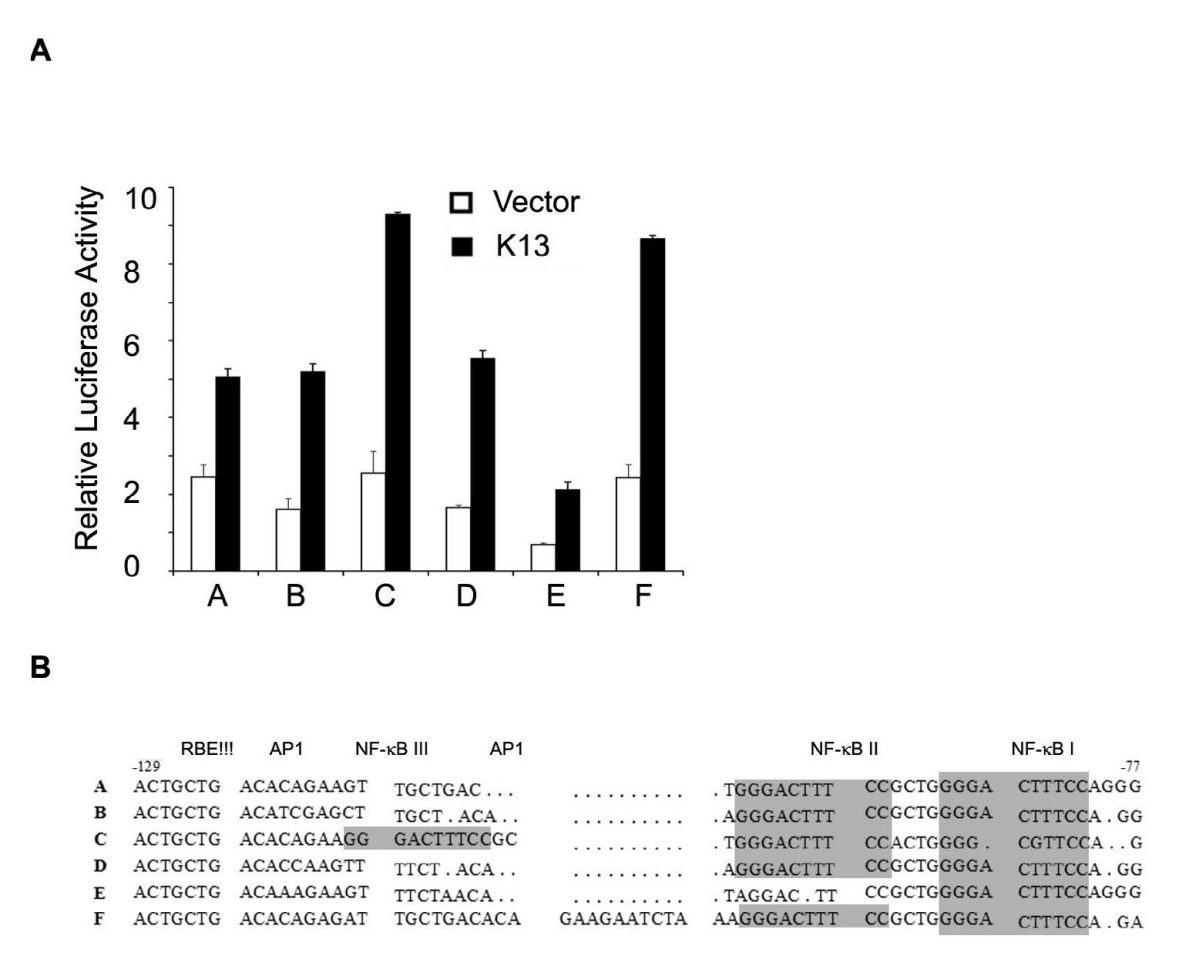
**Differential activation of HIV-1 subtype LTRs by K13**. A. 293T cells were transfected with an empty vector (pCDNA3) or K13 along with luciferase reporter constructs containing LTRs derived from the indicated strains of HIV and a β-galactosidase reporter construct as described in Fig. 1A. The values shown are averages (Mean ± S.E.) of one representative experiment out of three in which each transfection was performed in duplicate. B. Partial sequence of LTRs of HIV-1 subtypes A through F. The LTR region spanning positions -129 to -77 of subtype A is shown at the top. The NF-κB binding motifs are shaded gray.

There is considerable sequence diversity among the HIV-1 isolates that comprise the current global pandemic and these can be grouped into several distinct subtypes or clades [37]. In particular, the LTRs of different subtypes show distinct enhancer-promoter configuration and vary in the sequence and number of binding sites for different transcription factor, including NF-κB [38,39]. Although different HIV-1 LTRs are transcriptionally active, they differ in the level of basal reporter activity [38,39]. In addition, different HIV-1 LTRs are known to show differential response to TNF-α treatment, which correlates with the number of NF-κB binding sites [38,39]. Therefore, we sought to determine whether vFLIP K13 will differentially activate luciferase reporter constructs driven by LTRs derived from different HIV strains. Consistent with the published studies [38,39], we observed considerable difference in the basal activities of different HIV-1 LTRs promoters when transfected into 293T cells along with an empty vector (Fig. 7A). More importantly, coexpression of vFLIP K13 led to differential activation of luciferase reporter constructs containing LTRs from different subtypes of HIV-1 (Fig. 7A). Thus, subtype C, which possesses three NF-κB binding sites showed the maximum increase in vFLIP-induced HIV-1 LTR reporter activity while subtype E, which possesses only one NF-κB binding site showed the lowest level of basal and vFLIP-induced HIV-1 LTR transcriptional activation (Fig. 7A,B). These results demonstrate that, similar to situation with TNFα, K13 may differentially activate LTRs derived from different strains of HIV-1, which correlate with their NF-κB binding sites.

## Discussion

Although co-infection with HHV-8 and HIV-1 is known to synergistically increase the incidence of KS, until recently intracellular interaction between HHV8 and HIV-1 has not received adequate attention under the assumption that these viruses infect distinct cell types. Thus, HHV8 is typically believed to infect B lymphocytes, epithelial cells, keratinocytes, KS tumor cells, and endothelial cells [40,41], while the predominant host cells for HIV-1 are CD4^+ ^T lymphocytes, dendritic cells, and mononuclear phagocytes [41,42]. However, as recently pointed out by Huang *et al*, several lines of evidence suggest that the above assumption may not be completely true and HHV8 and HIV-1 may, in fact, interact *in vivo *[41]. *First*, both HHV8 and HIV-1 can efficiently infect cells of monocyte/macrophage lineage, including dendritic cells [43,44]. *Second*, Moir *et al *have shown that induction of CD4 and CXCR4 on B cells by CD40 stimulation leads to an increased susceptibility of these cells to T-trophic HIV infection [45]. *Third*, HHV8-infected B cells can be infected by HIV-1 via a cell-cell pathway and such infected B cells can support productive HIV-1 replication [46]. Finally, the range of HHV8-susceptible cells *in vivo *is unclear at the present. Therefore, it stands to reason that HHV8 and HIV-1 genomes may co-exist in the same cells *in vivo *and reciprocally regulate the gene expression of each other. Support for the above hypothesis is provided by a recent study which demonstrated that co-culture of HIV-1-infected CD4+ T cells with HHV8-infected B cell lines resulted in increased HIV-1 replication [47].

With the goal of elucidating intracellular signaling interactions which could be potentially involved in the induction of HIV-1 replication by HHV-8, we carried out a detailed analysis of the effect of HHV8 vFLIP on HIV-1 LTR activation. Consistent with an earlier report, we observed that HHV8 vFLIP strongly activates HIV-1 LTR in an NF-κB-dependent fashion [48]. We further demonstrate that vFLIP K13 could activate HIV-1 LTR in both epithelial and human lymphoma cell lines, although the magnitude of stimulatory effect was more pronounced in the epithelial cells. A possible explanation for this difference may lie in the differential expression of proteins that could modulate the effect of K13 on NF-κB and/or HIV-1 LTR activation. As an example, we demonstrate that K13-induced HIV-1 LTR activation can be effectively blocked by Murr1, a recently identified inhibitor of the NF-κB pathway which is highly expressed in T cells [32]. However, alternative explanation, including difference in the transfection efficiency between different cell lines, could apply as well.

We have recently reported that vFLIP K13 can activate both the classical and alternate NF-κB pathways and, as such, we were interested in determining the relative contribution of these pathways to K13-induced HIV-1 LTR activation. Based on the following data, we believe that activation of HIV-1 LTR is mainly through the classical pathway. *First*, our gel super-shift assay demonstrated that NF-κB complexes formed by vFLIP expression were primarily composed of c-Rel, p50 and p65 subunits. *Second*, siRNA-mediated downregulation of c-Rel and p65 led to near complete inhibition of K13-induced HIV-1 LTR activation whereas silencing of RelB expression was without significant effect. *Third*, K13-induced HIV-1 LTR activation was completely inhibited by super-repressor form of IκBα, which primarily blocks the classical NF-κB pathway. *Finally*, while K13 activates the alternate NF-κB pathway independent of IKK2, it failed to activate the HIV-1 LTR in IKK2-deficient MEFs.

Based on some early gene-knockout studies, IKK1 was believed to be not involved in cytokine-induced activation of the classical NF-κB pathway [49-51]. In the present study, we have observed that, in addition to IKK2- and NEMO-deficient MEFs, K13-induced HIV-1 LTR activation was markedly reduced in IKK1-deficient MEFs as well. We believe that the above results with IKK1-deficient cells do not necessarily support the involvement of the alternate NF-κB pathway in K13-induced HIV-1 LTR activation for the following reasons. *First*, we have recently reported that K13-induced p65/50 DNA binding and NF-κB transcriptional activation is markedly reduced in IKK1-deficient MEFs [23] Thus, the reduced HIV-1 LTR activation in the IKK1-deficient cells observed in the current study is consistent with requirement for IKK1 in K13-induced classical NF-κB activation. *Second*, recent studies suggest that IKK1 may be involved in transcriptional activation of classical NF-κB responsive genes through its ability to phosphorylate histones and p65 [52-54]. Thus, taken together, our results demonstrate that K13 activates HIV-1 LTR through the activation of the classical NF-κB pathway, in which IKK1 plays a major role. Thus, selective inhibitors of IKK1 may have a role in blocking K13-induced HIV-1 LTR transcriptional activation. However, it is important to point out that while IKK1 may be uniquely important for K13-induced classical NF-κB activation pathway, maximal activation of this pathway via K13 relies on cooperative interaction between IKK1, IKK2 and NEMO.

The transcription of cellular and viral genes is regulated by structural and functional interactions among a number of transcriptional factors that act in concert. This is also known to be the case with HIV-1 LTR. Thus, while NF-κB plays a major role in the transcriptional activation of HIV-1, it requires synergistic interaction with a number of cellular and viral proteins for maximal stimulation of this activity [1]. Although NF-κB is known to interact with Sp1, Ets and NF-AT to activate HIV-1 LTR, cooperative interaction between NF-κB and Tat has received the most interaction in the literature [1,55]. Tat has been shown to act synergistically with PMA, PHA and Tax-induced NF-κB to activate the HIV-1 LTR [1,34,36,55]. Consistent with these previous studies, we demonstrate that although K13 can activate the HIV-1 LTR by itself, it functionally cooperates with Tat to synergistically activate transcription from HIV-1 LTR. HIV-1 infection itself is known to induce persistent NF-κB activation, which is probably mediated via Tat and Nef [56,57], and interacts in a positive-feedback manner with Tat to enhance HIV-1 replication. However, in the immediate post-integration period of the HIV-1 life-cycle, Tat is expressed at very low levels which may not be enough to effectively stimulate HIV-1 LTR activation. Therefore, it is conceivable that vFLIP K13 could amplify the activity of Tat via NF-κB activation and thus support enhanced HIV-1 replication during the early stages of HIV-1 infection or in cells which express Tat at suboptimal levels.

The human immunodeficiency virus has considerably diversified during its worldwide spread in the current pandemic and can be classified into several distinct subtypes [37]. Subtype B is predominant in North America and Europe, subtype E in Southeast Asia and subtype C in sub-Saharan Africa, respectively [58]. Previous studies have demonstrated that LTRs from HIV-1 subtypes B, C and E vary in number and binding sites for NF-κB in their enhancer elements [59]. Thus, subtype C isolates are known to contain three functional NF-κB binding sites, as compared to two such sites in the enhancer of the more commonly studied subtype B [59]. On the other hand, in the subtype E, one of the NF-κB-binding sites has been switched to a GABP site, resulting only one functional NF-κB site and gain of a new specificity [60]. Consistent with the above results, in the present study we demonstrate that vFLIP-induced HIV-1 LTR activation is strongest in subtype C and weakest in subtype E. Thus, the differential response of different HIV-1 LTRs to K13-induced transcriptional activation may be explained on the basis of number of functional NF-κB sites in their enhancer elements. Future studies should address the question whether co-infection with HHV8 has a differential effect on the replication and natural history of different HIV-1 subtypes.

## Methods

### Plasmids, cell lines and reagents

Plasmids containing pcDNA3-K13-Flag and pcDNA3-E8-Flag, pRSV/LacZ and 293T cells have been described previously [21]. An expression construct encoding Murr-1 was generated by RT-PCR using cDNA prepared from H460 cells as a template and subsequently cloned in pcDNA3 vector with a C-terminal HA tag. Luciferase reporter constructs containing LTRs derived from different strains of HIV-1 (pBlue3'LTR-Luc-A-F) were obtained from AIDS Research and Reference Reagent Program, Division of AIDS, NIAID, NIH from Drs. Reink Jeeninga and Ben Berkhout. Wild-type and mutant HIV-1 LTR reporter constructs [61] and expression constructs for HIV Tat were obtained from Dr. Richard Gaynor. The IKKα-/- and IKKβ-/- mouse embryonic fibroblast cells were generated in Dr. Inder Verma's laboratory [62,63] and IKKγ/NEMO-/- cells were generated in Dr. Michael Karin's laboratory [64]. These cells were kindly provided by Dr. Richard Gaynor and were maintained in DMEM supplemented with 10% FBS. Jurkat cells were cultured in RPMI medium supplemented with 10% FBS and selected in the presence of 1500 μg/ml of G418 (Invitrogen). 293T cells were grown in DMEM with 10% FBS. Arsenic Acid was purchased from Sigma. MG132 and lactacystin were purchased from Calbiochem and Biomol, respectively. Retrovirus constructs containing C-terminal Flag epitope tagged HHV8 vFLIP (K13-Flag) was generated in MSCV neo-based retroviral vector and amphotropic viruses generated and used for infection as described previously [22].

### Electrophoretic mobility shift assay

Electrophoretic mobility shift assay was performed essentially as described previously [22], except an HIV LTR oligonucleotide duplex (sense strand, 5' TGC TAC AAG GGA CTT TCC GCT GGG GAC TTT CCA GG 3') was used instead of κB binding oligonucleotide. Nuclear extracts were prepared from Jurkat cells stably expressing an empty vector or vFLIP K13, which have been described previously [23]. Antibodies against p50, p65, RelB and c-Rel were purchased from Santa Cruz Biotechnology. An antibody against p52 was purchased from Upstate biotechnology

### Luciferase reporter assay

293 T cells were transfected in duplicate in a 24-well plate with the various test plasmids along with an HIV LTR/luciferase reporter construction (10 ng/well) and a pRSV/LacZ (β-galactosidase) reporter construct (75 ng/well) using calcium phosphate transfection protocol as described previously [21]. Cells were lysed 36–48 hours later and extracts were used for the measurement of firefly luciferase and galactosidase activity. Luciferase activity was normalized relative to the galactosidase activity to control for the difference in the transfection efficiency. Cos-7, Jurkat and MEF cells were transiently transfected with empty vector (pCDNA3) or K13 (500 ng/well) along with a HIV/luciferase reporter construct (100 ng/well) and a synthetic Renilla luciferase (phRL-TK; Promega) reporter vector (75 ng/well) by using LIPOFECTAMINE 2000 Reagent (Invitrogen, Carlsbad, CA) according to manufacturer's instruction. Thirty-six hours after transfection, cells lysates used for reporter assays. Luciferase activity was normalized relative to the Renilla luciferase activity to control for the difference in the transfection efficiency. The values shown are averages (Mean ± S.E.) of one representative experiment out of three in which each transfection was performed in duplicate.

### siRNA Oligonucleotides

siRNA oligonucleotides with two thymidine residues (dTdT) at the 3'-end of the sequence were designed to p65 (sense, 5'-GCCCUAUCCCUUUACGUCAdTdT-3'), c-Rel (sense, 5'-CAACCGUGCUCCAAAUACU dTdT-3'), RelB (sense, AGAUCAUCGACGAGUACAUdTdT-3') and control (sense, 5' GCGCGCUUUGUAGGAUUCGdTdT-3'), along with their corresponding antisense oligonucleotides. The RNA oligonucleotides were synthesized at RNA Oligonucleotide Synthesis Core facility, UT Southwestern Medical center. siRNA oligonucleotides (80 nM) were transfected using calcium phosphate as described previously [65].

### Western Blot

Western blot analysis was performed essentially as described previously [22]. Primary antibodies used in these experiments were: p65, c-Rel, Rel-B (rabbit polyclonal, Santa Cruz biotechnology) and actin (mouse monoclonal, Sigma).

## List of Abbreviations Used

DED, death effector domain; EMSA, electrophoretic mobility shift assay; FLICE, Fas-associated death domain-like IL-1 beta-converting enzyme; FLIP, FLICE inhibitory protein; HHV8, Human herpes virus 8; HIV-1, human immunodeficiency virus 1; KS, Kaposi's sarcoma; NEMO, NF-κB essential modulator; NIK, NF-κB-inducing kinase; NF-κB, Nuclear factor kappa B; MEF, murine embryonic fibroblast; PEL, primary effusion lymphoma, TNFR, Tumor necrosis factor receptor; IκB, inhibitor of NF-κB, IKK, IκB kinase; vFLIP, viral FLICE inhibitory protein; LTR, long terminal repeat.

## Competing Interests

The author(s) declare that they have no competing interests.

## Authors' contributions

QS and HM carried out most of the experiments described in this manuscript. PMC conceived of the study and participated in its design and coordination and helped to draft the manuscript. All authors read and approved the final manuscript.
